# The urologic epithelial stem cell database (UESC) – a web tool for cell type-specific gene expression and immunohistochemistry images of the prostate and bladder

**DOI:** 10.1186/1471-2490-7-19

**Published:** 2007-12-11

**Authors:** Laura E Pascal, Eric W Deutsch, David S Campbell, Martin Korb, Lawrence D True, Alvin Y Liu

**Affiliations:** 1 Department of Urology, and the Institute for Stem Cell and Regenerative Medicine, University of Washington, Seattle WA 98195, USA; 2 Institute for Systems Biology, Seattle WA 98103, USA; 3 Department of Pathology, University of Washington, Seattle WA 98195, USA

## Abstract

**Background:**

Public databases are crucial for analysis of high-dimensional gene and protein expression data. The Urologic Epithelial Stem Cells (UESC) database  is a public database that contains gene and protein information for the major cell types of the prostate, prostate cancer cell lines, and a cancer cell type isolated from a primary tumor. Similarly, such information is available for urinary bladder cell types.

**Description:**

Two major data types were archived in the database, protein abundance localization data from immunohistochemistry images, and transcript abundance data principally from DNA microarray analysis. Data results were organized in modules that were made to operate independently but built upon a core functionality. Gene array data and immunostaining images for human and mouse prostate and bladder were made available for interrogation. Data analysis capabilities include: (1) CD (cluster designation) cell surface protein data. For each cluster designation molecule, a data summary allows easy retrieval of images (at multiple magnifications). (2) Microarray data. Single gene or batch search can be initiated with Affymetrix Probeset ID, Gene Name, or Accession Number together with options of coalescing probesets and/or replicates.

**Conclusion:**

Databases are invaluable for biomedical research, and their utility depends on data quality and user friendliness. UESC provides for database queries and tools to examine cell type-specific gene expression (normal vs. cancer), whereas most other databases contain only whole tissue expression datasets. The UESC database provides a valuable tool in the analysis of differential gene expression in prostate cancer genes in cancer progression.

## Background

Public databases for the storage and retrieval of genomic and proteomic data have become an integral component of biomedical research. These databases can aid in the identification of genes and proteins responsible for disease and health and defining their function by enabling investigators in diverse research areas and interests with a range of computer expertise to have ready access to the stored information through one user interface. Previously, the Prostate Expression Database (PEDB) established a centralized archive of gene expression information for human prostate [1]. This database contains a large cDNA library of gene sequences obtained for normal/benign, benign prostatic hyperplasia (BPH), prostatic intraepithelial neoplasia (PIN) and malignant prostate disease states. The Prostate Gene Database (PGDB) is another prostate database that stores factual data about genes related to the human prostate and prostatic diseases supported by literature references [2]. These genes are grouped under molecular events of amplification, mutation, gross deletion, methylation, polymorphism, overexpression and linkage. These two databases provide valuable information obtained from whole prostate tissue. The characterization of tissues based on cell-surface protein expression [3] allows the possibility of separating cells of interest from that tissue for gene array analysis and determination of cell-type specific transcriptomes [4]. Public availability of cell-type specific data will be an important additional tool in future studies.

The Stem Cell Genome Anatomy Project (SCGAP) initiated by the National Institute of Diabetes and Digestive and Kidney Diseases (NIDDK) included seven organ-specific groups that were funded to form a research consortium. The aims of this consortium were to collectively develop necessary biological procedures and reagents for characterization of tissue specific progenitor cells and to characterize gene expression patterns in these cells using advanced technologies and bioinformatic techniques. The official web portal for SCGAP [5] was designed to deliver an overview of the progress of the consortium's research efforts and to function as a gateway to the websites of the consortium participants. As such, the detailed data, protocols and descriptions are accessible from the respective website of the participating SCGAP projects.

Our group, the urologic epithelial stem cells project, investigated the molecular basis of the differentiation of epithelial cells of the human prostate and bladder. We are interested in studying development and the cancer process in the context of interaction between individual cell types. Expression levels of CD cell surface antigens was first used to distinguish the constituent cell types of the prostate, as well as cancer cells from their normal counterpart [3,6]. The cell CD phenotyping data acquisition involved immunohistochemistry with ~200 commercially available CD monoclonal antibodies (BD-PharMingen). Magnetic cell sorting (MACS) based on the cell type-specific CD expression was then used to isolate the following prostatic cell types: CD31^+ ^endothelial cells, CD26^+ ^luminal secretory and CD104^+ ^basal cells of the epithelium, and CD49a^+ ^fibromuscular cells of the interglandular stroma for transcriptome profiling [4]. In addition, a CDw338+ (ABCG2) stem cell population was profiled [7]. These microarray datasets were also deposited in the UESC database [8]. Here, we will illustrate the utility of our UESC database, and a future consortium report will describe in detail the central SCGAP site and its federated search and data analysis tools.

## Construction and Content

The UESC database was based on the Systems Biology Experiment Analysis Management System (SBEAMS) [9], a software and database framework for collecting, storing, and accessing different types of experimental data. SBEAMS combined a relational database management system (RDBMS) back-end, a collection of tools to store, manage, and query experimental information and results, a web front-end for querying the database and providing integrated access to remote data sources, and an interface to other data processing and analysis programs. Since all data from each part of any experiment were organized in a modular schema using similar designs, quality control, analysis, and data integration tasks were greatly simplified. In SBEAMS, each module was made to operate independently but was built upon a core functionality, which included user authentication and auditing, web interface tools, result set management, Gene Ontology integration, centralized BioSequenceSet linking, RDBMS-independence layer, and others. Support for microarrays, proteomics, molecular interactions, macroarrays, gene expression localization, protein functional predictions, and expressed sequence tag (EST) clustering was provided in the current major modules. The SBEAMS module queries were automatically piped to Cytoscape [10] for network visualization and further exploration. Data includes cell type specific information for human and mouse prostate and bladder from immunostaining and microarray. Feedback options and data availability questions are made accessible through contact information listed on the website or through a feedback form.

### Populating the database

The methods of tissue collection, immunostaining and expression data used in this database have been published previously [3,4,11]. Briefly, tissue samples consisted of both cancer-enriched and cancer-free samples obtained from over 50 radical prostatectomies or cystectomies under approval by the University of Washington Institutional Review Board following a standard protocol.

### Immunohistochemistry data

The immunohistochemistry data for human and mouse prostate and bladder were annotated and uploaded in the database following the data standard, Minimum Information Specification For In Situ Hybridization and Immunohistochemistry Experiments (MISFISHIE) [11]. The files are systematically named based on the antibody used in staining, the organism, tissue type, tissue block, and magnification. As an example, the file 'CD44 98-395F HP ba 100.jpg' was stained with anti-CD44, was derived from human prostate tissue block 98-395F, is human prostate tissue, and an image of microscopic field of view b within field a was captured at 100× magnification. Annotation describes the tissue, distribution of reaction product in the tissue, distribution of reaction product in the tissue, localization patterns within histologic cell types, and provides an assessment of the level of expression of the protein for the immunostaining data. Data is available for ~200 CD antibodies for human prostate and bladder and ~20 CD antibodies for mouse prostate and bladder.

### Microarray data

#### Affymetrix array analysis of prostate cancer cell lines

The prostate cell transcriptomes of CD26^+ ^luminal epithelial, CD104^+ ^basal epithelial, CD49a^+ ^stromal fibromuscular, plus CD31^+ ^endothelial, CDw338^+ ^stem, and side population (SP) are all available in UESC [4,7]. To date, prostate cancer transcriptomes include those of lineage-related cancer cell lines LNCaP, C4-2, and CL1, those of PC-3 and DU145, plus that of a CD26^+ ^cancer cell type sorted from a primary tumor of Gleason 3+3. The bladder transcriptome data includes 1 replicate each of CD13^+ ^stromal and CD13^- ^stromal cells representing two sub-domains of the bladder lamina propria [12]. In the data module, the CEL files are the raw data from the array scan, the RPT files hold the statistical analysis of chip signals, the XML files are MAGE-ML descriptions of the experiments, and the Image files are synthetic JPEG images of the Affymetrix HG-U133_Plus_2 GeneChips.

#### MPSS analysis of prostate cancer cell lines

Results from MPSS (Massively Parallel Signature Sequencing) experiments on the LNCaP and C4-2 cell lines are also available in the UESC database [13]. Data may be downloaded as an Excel spreadsheet for each cell type, containing the accession number and experimentally detected TPM (transcripts per million) for each analyzed sequence. This data was used to compare MPSS with Affymetrix arrays in their coverage overlap [13].

### Searching the database

#### CD immunohistochemistry images

**Figure 1:**
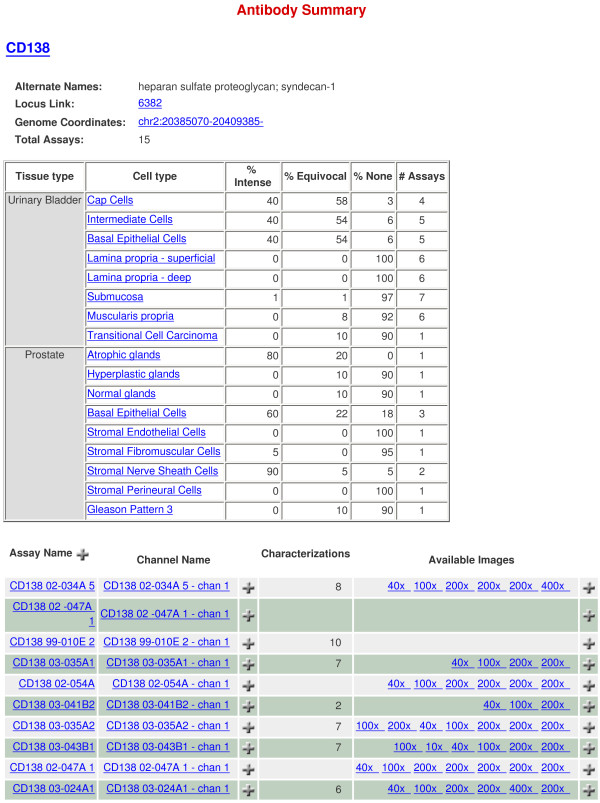
**CD immunohistochemistry**. Shown is the image data summary for CD138 (SDC1). The top table provides annotation data including the tissue type, distribution of reaction product in the tissue, localization pattern within histologic cell types and an assessment of the level of protein expression for the immunostaining data. The bottom table provides links to the available images for each annotated sample. Available immunostaining images and additional data can be retrieved by clicking on the links.

**Figure 2:**
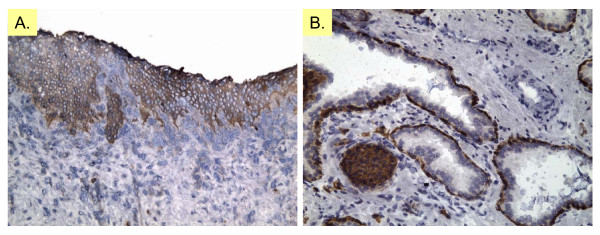
**CD138 immunostaining images**. CD138 (SDC1) immunoreactivity of normal human prostate and bladder (immunoreaction product red-brown; pale blue hematoxylin nuclear counterstain). (**A**) CD138 staining of human bladder urothelium, assay name CD138 03-035A1. (**B**) CD138 staining of human prostate atrophic glands, basal epithelial cells and nerve sheath cells, assay name CD138 02-007C 5. Original magnification is 200×.

The CD immunohistochemistry images may be viewed or downloaded as ZIP archives. Additionally, a summary of staining for various species and cell types can be downloaded directly as tab-delimited text files. Fig. 1 shows the data summary for CD138 (syndecan-1). Cell type-specific expression is scored by staining intensity, and the uploaded images (at multiple magnifications) of different tissue sections can be opened for examination. Fig. 2 shows CD138 staining at 200× magnification for human bladder and prostate.

#### Transcriptome data

The available array datasets (usually after accepted for publication) are listed and can be chosen for interrogation.

**Figure 3:**
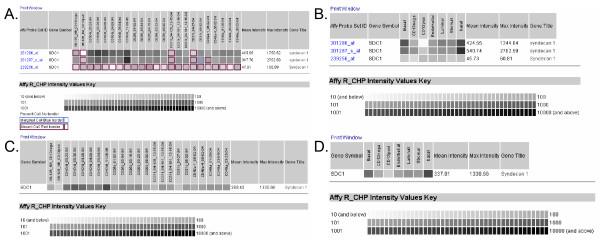
**Single gene search**. The expression of CD138 (SDC1) among the four sorted prostate cell populations (CD104^+ ^basal epithelial, CD26^+ ^luminal epithelial, CD31^+ ^endothelial and CD31^+ ^stromal fibromuscular) in addition to sorted bladder cell populations (CD13^- ^and CD13^+ ^bladder lamina propria) is illustrated. The Affymetrix signal intensity levels are represented by the grey scale. The data can be displayed in full (**A**), coalesced with respect to biological replicates (**B**), probesets (**C**), or both replicate and probeset (**D**). Present call boxes have no border, Absent calls have a red border, Marginal call is blue (not shown in this example).

**Figure 4:**
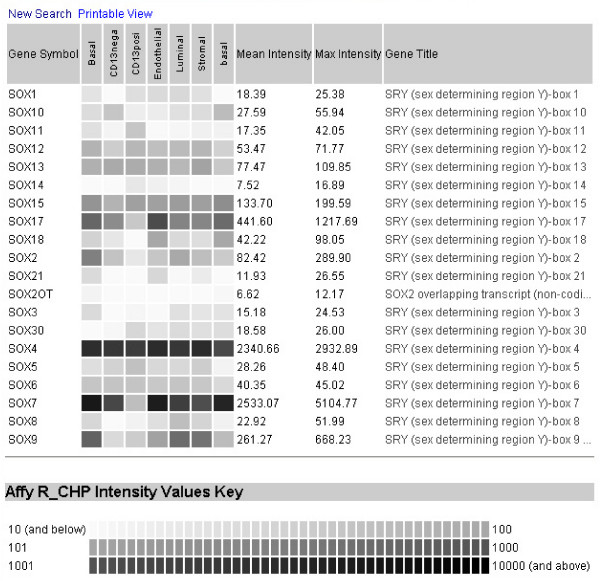
**Multiple gene search**. Shown is the query output for gene names containing SOX (sex determining region Y box) among the four sorted prostate cell populations (CD104^+ ^basal epithelial, CD26^+ ^luminal epithelial, CD31^+ ^endothelial and CD31^+ ^stromal fibromuscular) in addition to sorted bladder cell populations (CD13^- ^and CD13^+ ^bladder lamina propria).

(1) Single gene search – in which one can enter Affymetrix Probe Set ID, Gene Name, or Accession Number together with the options of coalescing probesets and replicates. In the Affymetrix HG-U133 arrays often times genes would be represented by multiple probesets, of which not all would give meaningful results. The hybridization signals for all probesets of one gene can be combined if the COALESCE PROBESETS tick box is clicked. The tick box COALESCE REPLICATES averages the signal for each of the biological replicates that make up a sort. The greyscale gradient indicates RMA normalized Affymetrix signal intensity. Signals of 10 or less are represented as white and signals greater than or equal to 10,000 are represented as black. Higher Affymetrix signal (more black) indicates higher levels of gene expression. Fig. 3 shows the analysis output for CD138 (SDC1). Fig. 3A shows the signal intensities scored by the three probesets for SDC1 of all replicates (n = 5) of four prostate cell types (CD104^+ ^basal, CD26^+ ^luminal, CD31^+ ^endothelial, CD49a^+ ^stromal) and one replicate each of two bladder cell types (CD13^- ^stromal and CD13^+ ^stromal) queried. CD138 expression is detectable in prostate basal cells, and lowered or undetectable in luminal, endothelial and stromal cells. As an illustration of probe variability, the 239256 probeset scored no expression or Absent Call in basal cells (5/5 replicates), and other cell types (5/5 replicates). Fig. 3B shows the analysis summary of CD138 cell-type expression coalesced by replicate, 3C coalesced by probeset, and 3D coalesced by replicate and probeset. This expression data is in accordance with the pattern of prostatic CD138 expression scored by immunohistochemistry (Fig. 1). Included in this summary is the level of CD138 expression in basal urothelial cells.

(2) Multiple gene (batch) search – in which searches can be initiated by using "%" as a wildcard character (e.g., CD% to list all official gene names with the CD designation) ["_" is a single character wildcard such that, e.g., A_ brings up AR; A__ brings up A2M, ABO to AXL, A___ brings up A1BG to ASB8, etc.]. Fig. 4 shows the query output for gene names with SOX% (sex determining region Y box). Other batch search examples would be IL (interleukin), ITG (integrin), TNF (tumor necrosis factor), ADAM (a disintegrin and metalloprotease domain) genes. This query feature is not widely available in many other databases.

## Utility and Discussion

UESC data types were organized in separate modules to afford a good balance between flexibility and consistency. The management system was designed to allow efficient data access to all levels of users, with both easy web and scriptable, sophisticated interfaces, and to be reusable so that a new project may be built on a previous one (e.g., kidney and bladder cancer data to prostate cancer data). The database will therefore continuously expand as more cell type-specific information becomes available. The UESC database will be a valuable tool in the analysis of differential gene expression in prostate cancer genes in cancer progression.

## Conclusion

Strategies for the analysis of the interface between gene expression and protein information involve a variety of computational methods that require the storage and retrieval of large datasets. These databases become perforce an integral component of biomedical research. The UESC database is a unique, web-accessible, searchable compilation of published data concerning the identification and characterization of genes and proteins in specific cell types of the urologic organs where cancer is a major disease. These cell populations retain to a high degree their CD phenotype as determined by immunostaining in intact tissue; concordance between gene expression measured by DNA array and immunohistochemistry was good and will be published separately. These cell type transcriptomes allow us to pursue many studies which are not possible with whole tissue transcriptomes.

## Availability and requirements

The UESC database is freely accessible at . It has been tested to work with Mozilla Firefox and Internet Explorer.

## List of abbreviations used

BPH: Benign prostatic hyperplasia

CD: Cluster designation

EST: Expressed sequence tag

MACS: Magnetic cell sorting

MISFISHIE: Minimum Information Specification For In Situ Hybridization and Immunohistochemistry Experiments

MPSS: Massively Parallel Signature Sequencing

NIDDK: National Institute of Diabetes and Digestive and Kidney Diseases

PEDB: Prostate Expression Database

PGDB: Prostate Gene Database

PIN: Prostatic intraepithelial neoplasia

RDBMS: Relational database management system

RMA: Robust multi-array average

SBEAMS: Systems Biology Experiment Analysis Management System

SCGAP: Stem Cell Genome Anatomy Project

TPM: Transcripts per million

UESC: Urologic Epithelial Stem Cell

## Competing interests

The author(s) declare that they have no competing interests.

## Authors' contributions

LEP drafted the manuscript with input from DSC, EWD and AYL. Database design and programming was performed by EWD, DSC and MK with input from AYL, LEP and LDT. LDT provided immunohistochemistry data annotation and staining summaries. All authors have read and approved the final manuscript.

## Pre-publication history

The pre-publication history for this paper can be accessed here:


